# Hydrogen-Fueled Microbial Pathways in Biogas Upgrading Systems Revealed by Genome-Centric Metagenomics

**DOI:** 10.3389/fmicb.2018.01079

**Published:** 2018-05-28

**Authors:** Laura Treu, Stefano Campanaro, Panagiotis G. Kougias, Cristina Sartori, Ilaria Bassani, Irini Angelidaki

**Affiliations:** ^1^Department of Environmental Engineering, Technical University of Denmark, Kongens Lyngby, Denmark; ^2^Department of Biology, University of Padova, Padova, Italy; ^3^Department of Agronomy, Food Natural Resources Animals and Environment, University of Padova, Padova, Italy

**Keywords:** anaerobic digestion, biogas upgrade, mesophilic, thermophilic, metagenomics, methanogens, syntrophs, metabolic reconstruction

## Abstract

Biogas upgrading via carbon dioxide hydrogenation is an emerging technology for electrofuel production. The biomethanation efficiency is strongly dependent on a balanced microbial consortium, whose high- resolution characterization along with their functional potential and interactions are pivotal for process optimization. The present work is the first genome-centric metagenomic study on mesophilic and thermophilic biogas upgrading reactors aiming to define the metabolic profile of more than 200 uncultivated microbes involved in hydrogen assisted methanogenesis. The outcomes from predictive functional analyses were correlated with microbial abundance variations to clarify the effect of process parameters on the community. The operational temperature significantly influenced the microbial richness of the reactors, while the H_2_ addition distinctively alternated the abundance of the taxa. Two different *Methanoculleus* species (one mesophilic and one thermophilic) were identified as the main responsible ones for methane metabolism. Finally, it was demonstrated that the addition of H_2_ exerted a selective pressure on the concerted or syntrophic interactions of specific microbes functionally related to carbon fixation, propionate and butanoate metabolisms. Novel bacteria were identified as candidate syntrophic acetate oxidizers (e.g., *Tepidanaerobacter* sp. DTU063), while the addition of H_2_ favored the proliferation of potential homoacetogens (e.g., *Clostridia* sp. DTU183). Population genomes encoding genes of Wood-Ljungdahl pathway were mainly thermophilic, while propionate degraders were mostly identified at mesophilic conditions. Finally, putative syntrophic interactions were identified between microbes that have either versatile metabolic abilities or are obligate/facultative syntrophs.

## Introduction

Biogas is produced through a biologically mediated process, which is called Anaerobic Digestion (AD). This process is performed by hundreds of different bacterial and archaeal species, whose taxonomy remains partially unknown. The produced biogas is composed of CH_4_ (50–70%), CO_2_ (30–50%), and trace gases (i.e., hydrogen sulfide) (Kougias et al., 2017b). Biogas represents a versatile energy carrier, since it can be used to generate electricity and heat. Nowadays, technological advances have been made in the area of biogas purification and upgrading. These processes target to increase the methane content of the final output gas, reaching a concentration of more than 90%, expanding the exploitation possibilities of biogas (Angelidaki et al., 2018). Indeed, direct injection of biomethane into the national gas grid is allowed by upgrading the biogas quality to similar levels as the natural gas standards (Sun et al., 2015). There are several methods for biogas upgrading including physicochemical and biological technologies (Alibardi et al., 2017).

Recently, increased attention has been given to biological methanation of H_2_ and CO_2_ via hydrogenotrophic methanogenesis (Luo and Angelidaki, 2012; Díaz et al., 2015; Agneessens et al., 2017). Methanogenic archaea are located at the bottom level of AD food chain, meaning that their role is strictly specialized to generate CH_4_ using simple substrates, such as H_2_ and CO_2_, formate, methanol and acetate. During hydrogenotrophic methanogenesis, H_2_ is consumed by methanogens to catalyze CO_2_ and/or other simple organic molecules into their most reduced form, which is CH_4_ (Angelidaki et al., 2011)_._ In conventional biogas production systems, H_2_ is provided by syntrophic bacteria that oxidize organic compounds using protons as electron acceptors. This metabolic reaction in non-syntrophic conditions is energetically unfavorable. However, H_2_ consumption by methanogens reduces the H_2_ partial pressure allowing the reduction to be exergonic (Stams and Plugge, 2009). For this reason, the parameter “interspecies H_2_ transfer” is fundamental for the optimal functioning of the biogas microbiome. Obviously, in case of biological biogas upgrading, the injection of supplemental H_2_ amounts enhances the disruption of the existing microbiome's harmony resulting in a new consortium, which is more specialized in H_2_ utilization.

The comprehension of the AD microbiome lifestyle has mainly been extrapolated from research on culturable microbial species. Nevertheless, this approach does not provide a holistic overview of the process, which is able to bridge essential biological gaps. For that reason, a direct description and interpretation of the AD mechanism is limited. Moreover, the strong syntrophic relation among bacterial and archaeal species hampers potential cultivation techniques and therefore it becomes even more difficult to gain information on single microbial species. A representative example of this argument is the limited number of species involved in acetate/CO_2_ conversion (syntrophic acetate oxidizing bacteria-SAOB), having the genome sequence currently available at public databases. However, knowledge derived from the few available genomes, and analysis of their metabolic pathways, represents an important step to assess their functions and relations with other members of the AD microbiome (Stams and Plugge, 2009; Worm et al., 2014).

In the last few years high-throughput sequencing and novel bioinformatics approaches paved the way for the reconstruction of the so-called microbial population genomes (PG), a process resulting in the characterization of microbial genomes obtained without isolation and cultivation (Albertsen et al., 2013; Nielsen et al., 2014; Campanaro et al., 2016). These studies led to implementation of “genome-centric metagenomics,” which is a collection of strategies allowing the genomic reconstruction of microbial species from the scaffolds obtained after shotgun sequence assembly of the entire microbial community (Albertsen et al., 2013; Nielsen et al., 2014; Campanaro et al., 2016). Despite the fact that the PGs are not “real” genomes derived from sequencing of DNA extracted from pure cultures, they represent an important resource for defining the functional properties of the microbial species by means of gene annotation and pathways identification (Kanehisa et al., 2014). The *in silico* investigation focused on gene functions allows identification of metabolic pathways and, in context of anaerobic digestion, can be used to divide the microbes into four classes: hydrolytic, acidogenic, acetogenic and methanogenic. The recovery of the PGs from a microbiome and the role assignment are essential to study the species, which are more recalcitrant to cultivation, such as the syntrophic microorganisms.

The present study is a novel research assessing the microbiome of biological methanation systems using genome-centric metagenomics. The reconstructed population genomes of biogas upgrading microbial communities were employed to elucidate for the first time the metabolic profile of H_2_ assisted methanogenesis both at mesophilic and thermophilic operational conditions. By determining the changes in microbial abundance, before and after H_2_ addition, and by using a composite bioinformatics strategy to annotate the genes encoded in the PGs, it was possible to suggest the functional roles of the microbes and to shed light on the syntrophic interactions occurring during the biogas upgrading process.

## Materials and methods

### Biogas reactor configuration, operation, and samples collection

The setup consisted of a two-stage CSTR, with a total working volume of 3.5 L (1.5 and 2 L for primary and secondary stage, respectively). The configuration operated either at mesophilic (35°C) or thermophilic (55°C) conditions. The hydraulic retention times (HRTs) were set to 25 and 33 days for primary and secondary mesophilic reactors, and to 15 and 20 days for thermophilic reactors. The primary reactor of each system was treating cattle manure and was serving as a conventional biogas digester. The total and volatile solids concentrations of raw manure were 47.40 ± 1.86 and 34.56 ± 1.40 g/L, respectively, had a pH of 7.44, total Kjeldahl nitrogen content of 3.03 ± 0.10 g-N/L, ammonium nitrogen concentration equal to 2.07 ± 0.01 g-N/L, and finally volatile fatty acids content of 6.83 ± 0.48 g/L. The produced biogas together with the effluent digestate was subsequently introduced to the secondary reactor (i.e., biogas upgrading chamber) in which H_2_ was externally provided. An overview of reactors' operation is given in Table 1 and more details are provided by Bassani et al. (2015). In brief, total and volatile solids, total Kjeldahl nitrogen and ammonium nitrogen concentrations were measure according to Standard Methods for Examination of Water and Wastewater (American Public Health Association, 2005). The determination of the biogas composition was measured using a gas chromatograph (Mikrolab, Aarhus A/S, Denmark) equipped with a thermal conductivity detector (TCD) and the measurement of volatile fatty acid concentrations was performed using a gas chromatograph (Shimadzu GC-2010, Kyoto, Japan) equipped with a flame ionization detector (FID). The pH values were measured by a digital PHM210 pH meter connected to the Gel pH electrode (pHC3105-8; Radiometer analytical). As the upgrading process occurred in the secondary stage, genomic DNA was extracted only from the secondary reactors, as previously described (Bassani et al., 2015). In brief, a representative sample from each upgrading reactor (i.e., mesophilic and thermophilic) before and after H_2_ addition was obtained for microbial analysis. The samples were collected during steady reactor operation (i.e., after 3 HRTs) to ensure representative process performances and microbial community stabilities.

**Table 1:** Reactors' operation at steady state conditions before and after H_2_ addition.

	**Mesophilic**		**Thermophilic**	
	**Pre H_2_**	**Post H_2_**	**Pre H_2_**	**Post H_2_**
Temperature (°C)	35 ± 1	35 ± 1	55 ± 1	55 ± 1
CH_4_ yield (mL/gVS)	111 ± 24	168 ± 21	249 ± 27	359 ± 22
Biogas composition (%)				
CH_4_	69.7 ± 0.3	88.9 ± 2.4	67.1 ± 0.8	85.1 ± 3.7
CO_2_	30.3 ± 0.3	8.8 ± 3.2	32.9 ± 0.9	6.6 ± 0.9
H_2_	0	2.3 ± 1.8	0	8.3 ± 3.6
H_2_ consumption efficiency (%)	0	98.8 ± 1.2	0	92.4 ± 3.3
pH	7.73 ± 0.15	8.17 ± 0.13	7.89 ± 0.17	8.49 ± 0.04
VFA (mg/L)	90.10 ± 49.23	155.94 ± 31.94	284.76 ± 168.24	381.41 ± 73.59
Acetate (mg/L)	61.90 ± 8.20	143.60 ± 39.51	236.34 ± 169.47	320.55 ± 58.34
Propionate (mg/L)	1.48 ± 0.33	3.30 ± 2.66	7.87 ± 7.66	42.27 ± 12.89
iso-butyrate (mg/L)	0.02 ± 0.07	0.20 ± 0.29	1.99 ± 0.65	1.05 ± 1.53
Butyrate (mg/L)	2.38 ± 0.84	1.16 ± 1.26	4.40 ± 1.95	1.52 ± 1.65
iso-valerate (mg/L)	0.09 ± 0.19	/	1.40 ± 1.24	1.13 ± 1.67
Valerate (mg/L)	0.92 ± 1.75	3.95 ± 9.24	0.84 ± 1.39	14.52 ± 18.79
n-hexanoate (mg/L)	23.32 ± 44.20	0.11 ± 0.33	31.91 ± 57.16	0.36 ± 0.66

### Taxonomy assignment and coverage calculation

Sequence data used in this study can be found at Sequence Read Archive (SRA) under accession SRP058235 and whole metagenome assembly is at DDBJ/EMBL/GenBank under the accession number LSQX00000000. Metagenomic assembly and binning processes were obtained from a previous work (Treu et al., 2016b). Completeness and contamination of PGs were re-calculated using CheckM (v1.0.7) (Parks et al., 2015) and compared with the previous results. All the PGs selected for metabolic reconstruction had completeness higher than 50% and contamination lower than 20% in at least one result, while the remaining PGs were used only for taxonomy evaluation. A more complete taxonomic assignment of the selected PGs was newly determined in the present study using four independent methods (Supplementary Datas 1, 2). Method 1-The scaffolds of each PG were checked using PhyloPythia software as previously described (Patil et al., 2012; Treu et al., 2016b). Method 2-Phylophlan (Segata et al., 2013) used as input 400 broadly conserved proteins for each PG and extracted the phylogenetic signal. Method 3-the 16S rRNA gene (most of them full-length) were recovered from the correspondent scaffold and assigned to the PGs considering the results of the binning; 16S rRNA sequences were taxonomically assigned using BLASTN on the 16S rRNA sequences database (NCBI). Method 4-The Average Nucleotide Identity (ANI) was calculated by selecting for each PG all the protein-encoding nucleotide sequences and using them to perform a similarity search with BLAT (Kent, 2002). All the gene sequences of each individual genome deposited at NCBI microbial database were used as reference in the similarity search. To accelerate the comparison, only the most complete genome was identified for each species (from approximately 70,000 genomes deposited at NCBI) and used for pairwise ANI calculation. All the genomes not assigned at species level were added to this subset of genomes. For each BLAT output derived from a pairwise “genomic comparison,” the average similarity value and the number of the orthologous genes identified were determined using an in house developed perl script. An average nucleotide identity higher than 95% and at least 50% of the genes of the PG having a match in BLAT were considered as thresholds for classifying two genomes as belonging to the same species (Konstantinidis and Tiedje, 2005). The higher common taxonomic assignment between methods 1 and 2 was used to assign a name to the PGs. The third method was used to evaluate the consistency of the taxonomic assignment obtained from methods 1 and 2. The fourth method was used to check the presence of microbes belonging to the same species in the NCBI microbial genome database. In order to build the archaea phylogenetic tree (Supplementary Data 3), mcrA protein sequences were recovered from all the proteins predicted from 755 archaeal genomes deposited at the NCBI microbial genome database using hmmsearch (v3.1b1) (Eddy, 2011). Protein sequences were aligned using Clustal Omega (v2.1+lgpl) (Sievers et al., 2011) and concatenated for constructing the phylogenetic tree using FastTree (v2.1.7) (Price et al., 2010). Results were visualized and edited with Dendroscope (v1.4.0) (Huson and Scornavacca, 2012).

The abundance value of each PG in different reactors and in different conditions was directly retrieved from the scaffold coverage. These values were obtained by aligning high quality reads to the global assembly with bowtie2 (v2.2.4) (Langmead and Salzberg, 2012) and the alignment results were transformed in the corresponding coverage values by using BEDTools (v2.17.0). The final coverage was calculated by normalizing the data, using the sample with the lowest number of sequences as reference.

### *In silico* functional analysis

The gene finding was performed using Prodigal (v2.6.2) in metagenomic mode on all the scaffolds (Hyatt et al., 2012). Annotation of total protein-encoding genes was performed using reverse-position specific BLAST algorithm, with COG RPSBLAST database (Galperin et al., 2015) (*e* < 1e-5) and with ghostKOALA for KEGG database (Kanehisa et al., 2016). Results were linked to each PG considering the assignment of the scaffolds obtained from binning. For each PG, the number of genes present on each KEGG and COG pathway was calculated using in house developed perl scripts (Supplementary Datas 4, 5). Manual investigation of specific KEGG maps was performed by coloring the genes in the pathway maps using KEGG mapper. The scaffolds assigned to each PG were uploaded to the RAST server and automatically re-annotated using SEED and the RAST gene caller (Overbeek et al., 2014). Annotation results were downloaded and used to determine the number of genes present in each first and second level SEED category; calculation was performed using in house developed perl scripts (Supplementary Data 6). All data and bioinformatics procedures can be found at www.biogasmicrobiome.com. A random sampling process was repeated 1000 times with a perl script implementing the Perl “rand()” function in order to determine for each PG the enrichment in specific functional categories as previously described (Treu et al., 2014). To determine the significance level, the same number of proteins encoded on each PG were randomly collected from the entire assembly and assigned to the functional categories. Finally, the fraction of random samples, in which the number of genes assigned to a specific functional category was equal or higher than N was determined (where N is the number of genes assigned to the same functional category in the PG under evaluation). If this fraction was lower than the significance level (α = 0.05) the enrichment for a specific functional category was considered significant. Abundant microbes were defined when their coverage was higher than 5 in at least one condition.

### Statistical analyses

Statistical analyses were performed to detect the role of functional categories in the variations in abundance of AD microbial communities, due to the applied operational parameters. The dataset consisted in single record information about the abundance of each PG considering both KEGG and SEED databases (Supplementary Data 7). Four operational conditions were investigated: C1 (mesophilic pre-H_2_), C2 (mesophilic post-H_2_), C3 (thermophilic pre-H_2_), and C4 (thermophilic post-H_2_). Data were transformed into six pairwise comparisons, in order to be analyzed as six variables: (i) Mean thermo/meso = log[(C3+C4)/(C1+C2)]; (ii) Mean post/pre H_2_ = log[(C2+C4)/(C1+C3)]; (iii) Meso post/pre H_2_ = log (C2/C1); (iv) Thermo post/pre H_2_ = log (C4/C3); (v) Pre H_2_ thermo/meso = log(C3/C1); (vi) Post H_2_ thermo/meso = log (C4/C2). Each PG was characterized in relation to operational conditions by calculating whether its log abundance exceeded by 1 Standard Deviation (SD) (including 68% of values), 2 SD (95% of values) or 3 SD (99% of values) the general mean for each variable obtained considering the whole microbial population (i–vi). A clustering procedure was performed using the Beta-Flexible Clustering Method (Milligan, 1989) to distinguish different groups of PGs based on their abundance in thermophilic or mesophilic conditions [variable (i)]. Moreover, this difference was confirmed using a chi-square test statistics including odd ratios calculation (OR) with confidence intervals (CI) considering PGs with changes in abundances higher than 2-folds in the two temperature conditions (Supplementary Data 7).

To identify functional categories with a significant impact on the relative abundance of PGs in different operational conditions, a multiple regression analysis was performed on data using the REG Procedure of the commercially available software SAS v. 9.3 (SAS Institute, Cary, NC). The six variables (i–vi) were considered as traits, and the number of genes ascribed to each functional category as predictors in a series of analyses. Functional categories were separately considered to avoid problems due to shared variance and over-parameterization. The analysis was corrected according to the completeness of each PG by including this term as predictor. A threshold of *P* ≤ 0.05 was chosen for defining the statistical significance of the functional categories.

The datasets supporting the conclusions of this article are included within the article and its Supplementary Files and are also available in the repository http://www.biogasmicrobiome.com under the “SYMBIO project.”

## Results and discussion

The 235 population genomes (PGs) belonging to uncultivated species previously extracted from stable biogas upgrading systems and characterized from a taxonomical perspective (Treu et al., 2016b) were used in the present study. Only PGs with completeness higher than 50% and contamination lower than 20% were used for metabolic reconstruction and correlation of microbial abundance with process parameters. According to the standards for the Minimum Information about a Metagenome-Assembled Genome (MIMAG) (Bowers et al., 2017) regarding completeness and contamination, 155 PGs can be classified as “medium-quality draft” and 66 as “high-quality draft”. Moreover, it can be estimated that the recovered PGs represented approximately half of the total community, considering that 38% and 57% of the total reads could be mapped to the PGs in mesophilic and thermophilic reactors respectively. The unbalance in the percentage of aligned reads was partially due to difficulties of extracting the most abundant hydrogenotrophic archaeal PG from the mesophilic reactor.

**Figure 1:**
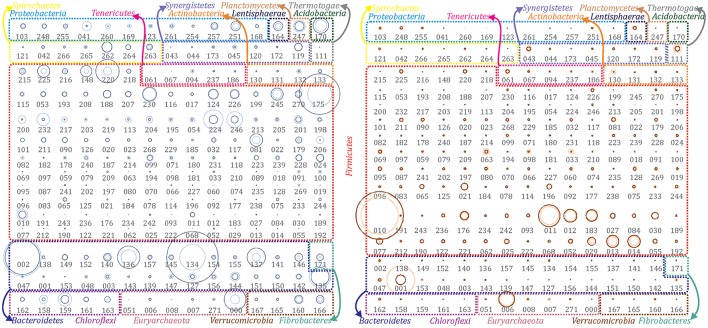
Abundance of extracted population genomes. All the reconstructed PGs were illustrated for both temperature conditions to emphasize the differences among the two communities. PGs abundance in the mesophilic **(left)** and thermophilic **(right)** biogas microbial community has been represented as bubble graphs. Abundance before (light blue/red) and after (dark blue/red) H_2_ addition are represented as circles. The area size of each circle is proportional to the abundance of the corresponding PG. Taxonomic assignment at phylum level has been indicated using colored borders and coloring phyla names accordingly. Numbers below circles refer to the IDs of the corresponding PG (e.g., DTU000).

Bioinformatic functional prediction analysis was applied in order to understand the effect of operational parameters on microbes and to assign their potential roles within AD process during biogas upgrade. Although most of the reconstructed PGs were identified at both temperature conditions (Figure 1), the overall community structure differed significantly (clustering analysis, *R*^2^ = 0.672) as evidenced by the microbial abundance profile. This comparison clearly demonstrated the existence of two communities responsible for the biomethanation: one mesophilic and one thermophilic (i.e., 35 or 55°C) (Figure 1, Supplementary Datas 1, 2). This separation was confirmed by calculating the standard deviations of single PGs from the mean abundance value of the whole community and indicated that 39% of them were higher abundant in mesophilic condition (more than 2-fold change), while 45% in thermophilic.

Most of the microbial species populating biogas reactors treating agro-industrial wastes were previously found to belong to three dominant phyla: *Firmicutes, Bacteroidetes* and *Proteobacteria* (Sundberg et al., 2013; Campanaro et al., 2016). However, particular attention needs to be given to infrequent phyla that are not members of “core AD microbiome” (Treu et al., 2016b), and to the fact that some phyla have high PG diversity, while others are represented only by few different species. For that reason, a profiling of the microbial communities was performed considering that the phyla can be classified as “oligo-member” (i.e., phyla that contain <5 different PGs) and “poly-member” (i.e., phyla that contain more than five different PGs). This grouping is useful to highlight major findings, such as that some PGs belonging to the “oligo-member” phyla were highly abundant, and thus had a particular role in the AD process (Figure 1 and Supplementary Figure 1).

Out of the 235 reconstructed PGs, 17% of them (i.e., 39 PGs) are the most abundant microbes of the biogas community. A recent study highlighted the direct correlation between microbial abundance and activity (Treu et al., 2016a); thus, the current finding agrees the extrapolation of Pareto axiom to the AD microcosm. In fact, according to the “Pareto 80/20 principle,” <20% of the total community is contributing to 80% of the overall performance (Verstraete et al., 2007). However, there is still an open debate on this concept and more investigations will be needed to extend it to a broader range of ecosystems or conditions.

### General metabolic insights into the microbial communities

*Acidobacteria, Fibrobacteres* and *Planctomycetes* were among the “oligo-member” phyla of the mesophilic community. Each phylum was represented by only one PG. *Chloroflexi* was another “oligo-member” phylum, which included five different PGs (Figure 1 and Supplementary Figure 1). It is worth mentioning that *Chloroflexi* spp. were exclusively identified at mesophilic conditions. This result indicates their inability to survive during thermophilic reactor operation. The predominance of *Chloroflexi* at mesophilic temperature was already reported in biogas reactors treating household waste (Levén et al., 2007). *Chloroflexi* sp. DTU159 was the only highly abundant microbe of this phylum and was negatively affected by exposure to increased H_2_ concentration (Supplementary Data 2). DTU159 presents a higher number of genes related to hydrolysis of polymers (galactose and starch) and H_2_ production compared to the other PGs. Moreover, it encodes a very high number of proteins related to envelope stress response, drug transporters (multidrug resistance, efflux pump) and toluene degradation (to benzoyl-CoA; Supplementary Data 4). The latter is in agreement with previous findings reporting that *Chloroflexi* are members of anaerobic community known to degrade toluene (Ficker et al., 1999). *Acidobacteria* sp. DTU170 was detected as one of the most abundant microbes, whose coverage was slightly increased after H_2_ addition (2-fold). This PG can be included in the first steps of the AD process, “hydrolysis” and “acidogenesis,” as it possesses the highest number of genes assigned to several KEGG categories related to protein degradation (i.e., amino acid utilization and reduction of sulfate to H_2_S; Supplementary Data 4). According to PhyloPhlAn, *Fibrobacteres* sp. DTU171 belongs to genus *Fibrobacter*, that is known to include species extremely active in polysaccharides degradation (Suen et al., 2011). The chemical composition of cattle manure, used as reactors' feedstock, is rich in polysaccharides considering also that it contains a significant amount of plant fibers derived from the animal's nutrition diet. Concordantly, DTU171 genome was found to harbor numerous genes involved in hydrolysis of polymers (i.e., cellulases; Supplementary Data 4). Even though it was detected in low abundance, this PG increased 30-fold after H_2_ addition (Figure 1 and Supplementary Data 2) and this behavior is consistent with the ability of *F. succinogenes* to use H_2_ as electron donor for fumarate reduction (Suen et al., 2011). The third “oligo-member” phylum includes only *Planctomycetia* sp. DTU247 (completeness 93%), having 90% similarity based on its 16S rRNA gene to *Thermogutta hypogea* (Slobodkina et al., 2015). *Planctomycetia* sp. DTU247 has the largest genome among all the reconstructed PGs in this study (nearly 6 Mbp; Supplementary Data 2). The large genome size (up to 10 Mbp) is a typical feature of *Planctomycetes* species. Previous research concluded that the large genome size was correlated with their adaptability to diverse environments because they are capable of degrading a wide range of biopolymers (Glöckner et al., 2003). Metabolic reconstruction of DTU247 genome confirms this argument and suggests a hydrolytic role of this microbe (Supplementary Data 4) due to the high number of genes involved in amino sugar metabolism (30) and in the two-component signal transduction systems (44). Moreover, DTU247 genome contains several genes involved in sulfur metabolism (12), inorganic (11) and organic (6) sulfur assimilation. In particular, DTU247 has genes for assimilatory sulfate reduction (${\text{SO}}_{4}^{-2}$), and more specifically encoding sulfatases (*cysND, cysC, cysH, PAPSS, sat, sir*) that reduce sulfate to sulfide (S^2−^) through sulfite (${\text{SO}}_{3}^{2-}$). Besides assimilatory sulfate (${\text{SO}}_{4}^{-2}$) reduction pathway to incorporate sulfur to aminoacids, sulfide (S^2−^) assimilation can potentially remove sulfur from biogas, as it is captured as microbial biomass. Therefore, it can be hypothesized that *Planctomycetia* sp. DTU247 has potential ability in chemotrophic desulfurization.

On the contrary, the “poly-member” phyla of the mesophilic microbial community were *Firmicutes* (41 PGs), *Bacteroidetes* (23 PGs) and *Proteobacteria* (nine PGs) (Figure 1 and Supplementary Figure 1). Emphasis is given to *Bacteroidetes* because of their remarkably higher diversity in mesophilic, compared to thermophilic conditions and due to the fact that five members of that phylum were among the most abundant in the mesophilic reactor (i.e., Mcov ~46). The majority of the PGs belonging to *Bacteroidetes* encode high number of carbohydrate active enzymes (CAZy; Supplementary Data 4) involved in polysaccharides degradation (e.g., *Bacteroidetes* sp. DTU142, *Bacteroidetes* sp. DTU149 and *Bacteroidales* sp. DTU136) indicating that they were the main degraders of organic matter. In particular, *Bacteroidetes* sp. DTU149 was found to have >99% Average Nucleotide Identity (ANI) with *Fermentimonas caenicola* str. ING2-E5B, isolated from a mesophilic CSTR co-digesting maize silage and manure (Hahnke et al., 2016), and able to ferment carbohydrates and complex proteinaceous substrates. Moreover, PGs assigned to *Bacteroidetes* had on average a lower number of genes related to “stress resistance” in comparison to other PGs and this can partially explain their prevalence at mesophilic conditions.

In the thermophilic reactor, *Actinobacteria, Synergistetes, Thermotogae*, and *Bacteroidetes* were identified among the “oligo-member” phyla. It is remarkable that the members of all these phyla were strongly inhibited after H_2_ addition as their abundance decreased by 2–20 fold. *Thermotogaceae* sp. DTU111 was found to be one of the most abundant microbes of thermophilic community. According to both ANI and 16S rRNA gene it was assigned as *Defluviitoga tunisiensis* L3 (98% identity). *D. tunisiensis* and *Thermotogae* phylum are known to play an important role in complex polysaccharides hydrolysis (Maus et al., 2015); *Thermotogales* were proposed to act synergistically with *Clostridiales* since the latter did not possess the β-sugar pathway (Xia et al., 2014). Concordantly, *Thermotogaceae* sp. DTU111 has a high number of genes in functional categories related to hydrolysis of polymers (e.g., starch) and CAZy. Moreover, genes belonging to amino acid utilization metabolism and sulfate-sulfur assimilation were abundant in this PG indicating potential hydrolytic activity on a wider range of substrates (Supplementary Data 4).

As it was previously discussed, the number of PGs assigned to *Bacteroidetes* phylum was extremely low under thermophilic conditions, while most PGs were assigned to *Firmicutes* (eight out of ten of the most abundant, Mcov ~81). Based on the metabolic reconstruction of the PGs, it was shown that the role of *Bacteroidetes* spp. at mesophilic conditions is taken over by *Firmicutes* spp. at thermophilic temperatures, indicating a functional redundancy of these two taxa. In fact, *Bacteroidetes* phylum was represented by only three PGs, one of which (*Rikenellaceae* sp. DTU001) was the second most abundant microbe of the community. DTU001 was further assigned to genus *Alistipes* and to the hydrolytic step of AD. Moreover, it was closely related to *Rikenellaceae* sp. DTU002 (dominant at mesophilic conditions) and was inhibited by H_2_ addition. A similar finding was related to genus *Methanoculleus*, suggesting that closely taxonomically related PGs can alternatively occupy the same ecological niche in different operational conditions. It should be noticed that only the accurate investigation of the microbiome provided by a genome-centric approach can perform a reliable characterization of single microorganisms. For instance, the aforementioned PGs (i.e., *Rikenellaceae* spp. and *Methanoculleus* spp.) could not have been distinguished from closely related species using a direct taxonomical assignment of the shotgun reads to a database of known microbial genomes.

Considering the “poly-member” *Firmicutes* phylum, the vast majority of the PGs (64 PGs) was assigned to *Clostridiales* order and mostly to *Syntrophomonadaceae* family (35 PGs) (Supplementary Data 2). Gene annotation and predictive functional reconstruction verified the role of *Syntrophomonadaceae* spp. as syntrophic fatty acid oxidizers, confirming previous findings related to similar microbes (Sousa et al., 2007; Treu et al., 2016a). More specifically, these PGs were ranked among the top 10 having the highest number of genes assigned to fatty acids β -oxidation category (Supplementary Data 4), including *Syntrophothermus* sp. DTU052 (41 genes), *Clostridia* sp. DTU183, DTU021 and DTU077 (>10 genes and candidate homoacetogens). The overall effect of H_2_ on the abundant *Clostridiales* was positive, even if the change was not remarkable (up to 2-fold). *Clostridiales* sp. DTU010 was the absolute dominant of the thermophilic AD community (up to 462 cov). Results of functional pathway reconstruction confirmed the role of population *Clostridiales* in the hydrolytic step of AD, highlighted by the presence of a high number of genes in categories: multiple sugar transporters, hydrolysis of polymers and amino acid metabolism (Supplementary Data 4). This outcome confirms the substitutional role of *Bacteroidetes* and *Firmicutes*, which is fundamental for the functional classification of AD food chain in mesophilic and thermophilic conditions. Additionally, the identification of formyltetrahydrofolate synthetase (*fhs*) and other two genes of the Wood-Ljungdahl (WL) pathway (i.e., *folD* and *fdhA*) in *Clostridiales* sp. DTU010, along with its increased abundance after H_2_ addition, suggest that this microbe can act as homoacetogen. However, considering the limited number of genes identified, further investigations are needed to identify the alternative genes involved in this potential functional activity.

### Effect of H_2_ addition on archaea and syntrophic bacteria

It is well-known that the main H_2_ producers and consumers are involved in the final steps of the AD food chain (Bassani et al., 2015). Therefore, the external provision of H_2_ will cause a perturbation on the equilibrium existing between the different microbial species and alter the intricate network of interactions established among the members of the microbial community. In the following subsections the metabolic reconstruction is focused on syntrophic associations and their relevant pathways (e.g., methanogenesis, propionate and butyrate degradation, Wood-Ljungdahl pathway).

**Figure 2:**
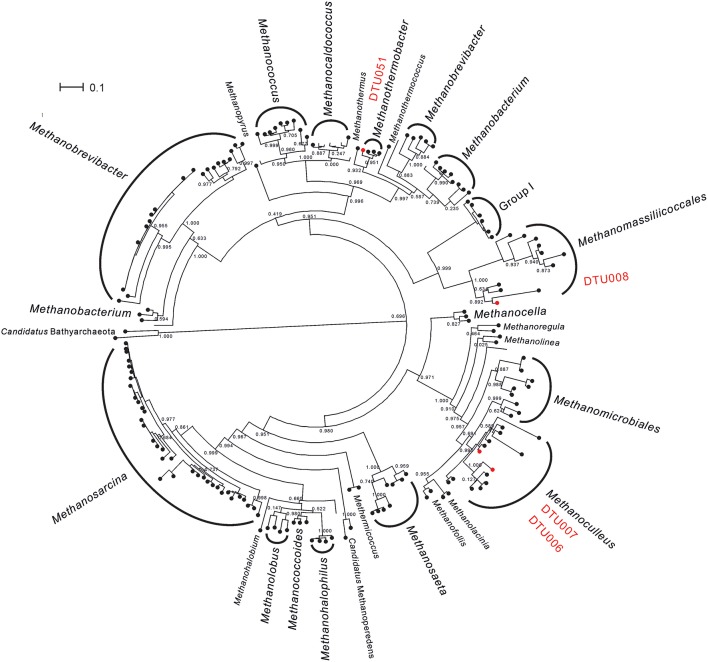
Phylogenetic relationships among archaea. Phylogenetic tree based on *mcrA* gene comprising all archaea with whole genome sequence deposited at NCBI. PGs considered in the present study and belonging to the thermophilic community are highlighted in red. *Methanosarcina* sp. DTU271 was not included since its *mcrA* gene was not recovered by the binning process.

The archaeal community of the upgrading systems was extremely simple and composed by only five PGs, all belonging to *Euryarchaeota* phylum (Supplementary Data 2). This is in agreement with previous studies performed in biogas reactors, in which the most frequently identified methanogens belonged to three main genera: *Methanoculleus, Methanothermobacter*, and *Methanosarcina* (Luo et al., 2015; Stolze et al., 2015). In addition to the analyses performed for all the PGs, the phylogeny of the archaea was investigated using mcrA protein (Figure 2 and Supplementary Data 3). The thermophilic community was dominated by the recently described species, *Candidatus* Methanoculleus thermohydrogenotrophicum DTU006 (Kougias et al., 2017a). In contrast, the mesophilic community was dominated by a different hydrogenotrophic *Methanoculleus* species (defined as “*Methanoculleus* spp. DTU000”) with similar abundance as the DTU006 genome. However, the quality of the reconstructed PG was not fulfilling the quality criteria set in the current study (i.e., it was probably a combination of two different species belonging to genus *Methanoculleus*; Supplementary Figure 2) and thus, it was excluded from further metabolic analysis. Other methanogenic archaea, such as *Methanoculleus thermophilus* DTU007, *Euryarchaeota* sp. DTU008 and *Methanothermobacter* sp. DTU051 were also present, mainly in the thermophilic community, but in very low abundance. The preference of *Euryarchaeota* sp. DTU008 to thermophilic temperature adds a new insight to the poorly described methanogen, which was recently assigned to *Methanomassiliicoccales* order (Campanaro et al., 2016). It is worth noting that the 16S rRNA gene of *Euryarchaeota* sp. DTU008 was similar to genus *Thermogymnomonas* (97% identity), which was previously found to be able to survive in acidic environment (Itoh et al., 2007). Moreover, *Methanosarcina mazei* DTU271 was observed only to a minor extent independently from the operational temperature. The effect of H_2_ on *Methanosarcina mazei* DTU271 was in agreement with previous findings showing an inhibition of the acetogenic methanogenesis in *Methanosarcina thermophila* TM-1 at high H_2_ partial pressure (Ahring et al., 1991). In contrast, it is surprising that hydrogenotrophic methanogens like *Methanoculleus thermophilus* DTU007 or *Methanothermobacter* sp. DTU051 decreased their abundance after H_2_ addition. However, it has to be considered that different hydrogenotrophic species are adapted to environments with markedly different H_2_ partial pressures, ranging from very low in sediments to very high when they obtain the gas from H_2_-producing fermentative microorganisms (Kramer and Conrad, 1993; Zinder, 1993). For this reason, an increased H_2_ concentration can sometimes determine a reduction in the abundance of some hydrogenotrophic archaea. Moreover, the extremely heterogeneous behavior of the archaeal PGs in response to the different operational parameters (i.e., increasing or decreasing in abundance and diversity of methanogenic species) could probably contribute to maintaining a mechanism for balancing CH_4_ production. The outcome indicates high functional redundancy in the archaeal community of such engineered ecosystems meaning that the role of a specific methanogen in the microbiome can be replaced by another microbe better adapted to the new condition. This is particularly evident for the dominant *Methanoculleus* PGs (DTU006 and DTU000), which share the same functional role at different operational conditions.

### Syntrophic acetate-oxidizing bacteria (SAOB)

The enormous number of bacterial species and the complexity of their metabolic properties normally hamper the investigation of “H_2_-producing” and “H_2_-utilizing” microbes. However, in the present study, the bioinformatic analyses provided the gene annotation of each microbial genome, and therefore, allowed the elucidation of syntrophic metabolic interactions occurring in the microbiome. Moreover, the low number of identified archaeal species assisted the investigation of their role as H_2_ scavengers in the reactors analyzed.

As previously mentioned, PGs encoding genes related to WL pathway, tricarboxylic acid (TCA) cycle, propionate and butyrate degradation were considered with great attention. Additionally, the carbon-monoxide dehydrogenase gene and genes for the generation of menaquinone cofactors were included in the analysis, since their corresponding proteins are involved in the transfer of H_2_ and formate from syntrophic bacteria (e.g., *Syntrophomonas wolfei*) to archaea (e.g., *Methanospirillum hungatei*) (Schink and Friedrich, 1994).

**Figure 3:**
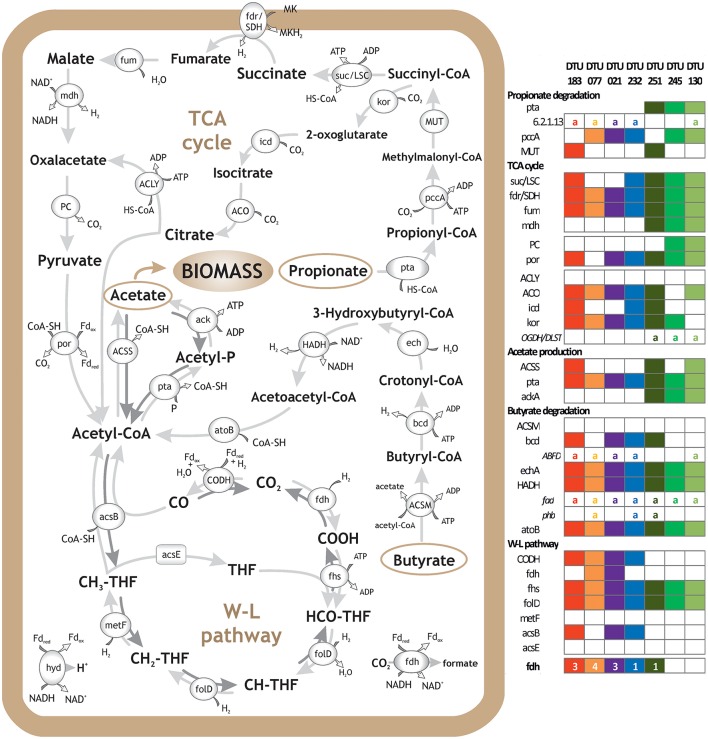
Metabolic reconstruction of syntrophic bacteria. Schematic representation of VFA degradation and H_2_ and CO_2_ utilization, including Propanoate and Butanoate degradation, TCA cycle and WL pathway. Genes' presence in selected PGs is reported in the right panel. The selection of the PGs was based on their relevance with the aforementioned pathways and importance for the upgrading process; “a” letter indicates alternative pathway and numbers indicate gene isoforms or subunits of the multiprotein complexes. *Clostridia* sp. DTU183 gene content is depicted in red (potential homoacetogen); *Clostridia* sp. DTU077 gene content is depicted in orange (potential homoacetogen); *Clostridia* sp. DTU021 gene content is depicted in purple (potential SAOB); *Syntrophomonadaceae* sp. DTU232 gene content is depicted in blue (potential SAOB); *Proteobacteria* sp. DTU251 gene content is depicted in dark green (potential propionate/butyrate degrader); *Firmicutes* sp. DTU245 gene content is depicted in green (potential propionate degrader); *Corynebacterium* sp. DTU130 gene content is depicted in light green (potential propionate/butyrate degrader).

Among putative syntrophs, 14 PGs had nearly complete (≥8 genes) WL pathway according to KEGG (Table 2) and were assigned to *Syntrophomonadaceae* (12 PGs), *Tepidanaerobacter* (DTU063) and to the order *Thermoplasmatales* (DTU008). *Tepidanaerobacter* sp. DTU063 was found to be abundant in the thermophilic reactor prior to H_2_ addition and was found to belong to genus *Tepidanaerobacter* according to PhyloPhlAn. We can thus hypothesize that it might have a role similar to the well-known SAOB *Tepidanaerobacter acetatoxydans* (Westerholm et al., 2011). It should be noted that DTU008 was the only *Euryarchaeota* harboring the genes of the WL pathway, and thus, a potential acetogenic activity for biomass production was hypothesized. It has to be considered that the presence of this gene cluster is not a *per se* demonstration of the microbial ability to follow the metabolic pathway in an oxidative (syntrophic acetate oxidation-SAO) or reductive (WL pathway) direction. It is known that some acetogens (often called homoacetogens), such as *Thermoanaerobacter kivui*, are unable to use this pathway in an oxidative way (Cord-Ruwisch et al., 1988; Weghoff and Müller, 2016). It should be underlined that only a few taxa found in this study were previously investigated at genomic level according to a comparison made with the publicly available microbial genomes. This finding is particularly important since uncharacterized SAOB and homoacetogens give the possibility to assess their unexplored functions. Another finding was that some of the PGs harboring a nearly complete WL pathway are able to perform other metabolic activities related to syntrophy. More specifically, six PGs had also a high number of genes involved in butyrate degradation and one (*Clostridia* sp. DTU095) had at least 20 genes related to propionate degradation (Table 2, Figure 3, and Supplementary Data 4). These PGs serve as syntrophic propionate or butyrate degraders together with other members of the *Syntrophobacteraceae* or *Syntrophomonadaceae* families. A combination of SAO and butyrate oxidation was reported previously (Hagen et al., 2017), while coupling with potential SAOB with propionate degradation was not observed before.

**Table 2:** PGs having genes involved in specific metabolic pathways found to be influenced by H_2_ addition.

**PG IDs**	**Thermo post/pre H_2_**	**Meso post/pre H_2_**	**Prop**	**But**	**CFP**	**FAD**	**WL**	**TCA**	**fdhF**	**ISA**	**Me**
DTU232	−4.65			24^*^		10^*^	8	14	1		
DTU204	−2.98			12^*^		18^*^	9	7	4		
DTU223	−0.17	−0.99					8	6	3		1
DTU183		0.43	14^*^	20^*^	32^*^	14^*^	8	11	3		
DTU077		4.04		14^*^		14^*^	10	8	4		
DTU122		2.15		1			8	11	5		
DTU192		0.73					8	5	3		
DTU236		0.64	18^*^	24^*^	29^*^		10	11	2		
DTU093		0.55		29^*^	39^*^		9	32	1		1
DTU027		0.24					8	9	2		
DTU021		−0.08		22^*^		21^*^	10	13	3		
DTU095		−0.44	23^*^	28^*^	62^*^		9	27	3		1
DTU063		−2.33			33^*^		8	22			
DTU008		−2.69	15^*^	15^*^	28^*^		8	15			
DTU251	1.35		27^*^	26^*^		21^*^		21	1	14	1
DTU245	0.92		20^*^			9		29			3
DTU254	−0.21		22^*^	14^*^		13^*^		20	1		
DTU156	−1.62		27^*^					24	2		7
DTU260	−4.63		22^*^	17^*^		17^*^		20		17	2
DTU041	−5.09		28^*^	41^*^		23^*^		28	1	14	6
DTU133	0.38	0.22	27^*^	25^*^		14^*^		26^*^			9
DTU101	0.10	−1.24	20^*^					24			
DTU249	−1.51	−1.26	30^*^	36^*^		28^*^		31^*^	1		3
DTU255	3.16	2.56	20	23^*^		13^*^		31^*^	4		5
DTU248	−0.66	0.58	17^*^	24^*^		10^*^		20	5	18	6
DTU037	−1.23	−2.53	30^*^	33^*^		17^*^		24	3	24	2
DTU258	−7.15	−8.22	21^*^			11^*^		15			
DTU131	−1.10	−3.81	28^*^	27^*^		19^*^		22^*^			9
DTU250	−1.64	−5.06	31^*^	25^*^		27^*^		23			6
DTU190		2.28	24^*^	45^*^		25^*^		15	8		4
DTU052		1.30	19^*^	35^*^	34^*^	38^*^		12	9		2
DTU030		0.11	16^*^	21^*^	44^*^			40	1		
DTU019		−1.38	30^*^	27^*^	38^*^			20	6		
DTU130		−4.66	23^*^	26^*^		13^*^		31^*^			9
DTU018		−5.21	20^*^	36^*^		17^*^		18	9		7

*In the upper part of the table are reported PGs potentially classified as SAOB or homoacetogens; in the lower part of the table are reported the syntrophic PGs (propionate and butyrate degraders). Asterisks indicate statistically significant number of genes in a specific PG in comparison with other genomes (1,000 random sampling process as explained in Methods section; p-values are reported in Supplementary Data 4). The presence of formate dehydrogenase (fdhF) gene in the PGs is also included. Abbreviations: Prop (propanoate metabolism), But (butanoate metabolism), CFP (carbon fixation pathways), FAD (fatty acids degradation), WL (Wood–Ljungdahl pathway), TCA (TCA cycle), fdh (formate dehydrogenase), ISA (inorganic sulfur assimilation), Me (menaquinone). In the second and third column are reported the ratio (log_2_) of the PGs abundance after H_2_ addition vs. before H_2_ addition in thermophilic (thermo) and mesophilic (meso) conditions*.

The vast majority of potential SAOB and homoacetogens were assigned to the thermophilic community and only two were mesophilic (Table 2). In fact, results showed that thermophilic PGs had a high number of genes related to cellular response to thermal stress, such as post-translational modification/protein turnover/chaperones (Supplementary Data 6), osmoprotectant transport system (Supplementary Data 4) and heat shock/oxidative stress (Supplementary Data 7). Thermophilic condition was also statistically correlated (SEED; *p* = 0.03) with the SEED category “hypothetical associated with RecF” protein (Table 3 and Supplementary Data 3). This protein has been recognized as part of the survival strategy in some extremophiles (e.g., in radiation-resistant microorganisms) (Singh and Gabani, 2011), and also involved in the pathway of the RecA protein acting in DNA repair as part of heat-shock response (Giliberti et al., 2002; van der Veen et al., 2007). The higher presence of putative thermophilic SAOB is in agreement with previous findings reporting that hydrogenotrophic methanogenesis via WL pathway is outcompeting acetoclastic methanogenesis, since these microbes are more resistant to stressful conditions (Schink, 1997).

**Table 3:** Statistical correlations between overall metabolic functional categories and operational parameters.

**Mesophilic vs. thermophilic condition**				**Before (pre) vs. after (post) H**_**2**_ **addition**			
**KEGG**							
	**Category**	**PrF**	**b**		**Category**	**PrF**	**b**
Thermo	Riboflavin m.	0.04	0.26	Post	Polyketide sugar unit b.	0.04	0.12
	Nitrotoluene degr.	0.03	0.18	Pre	Two-component sys.	0.03	−0.01
	Peptidoglycan b.	0.02	0.11		Flagellar assembly	0.05	−0.01
	Flagellar assembly/Bacterial chemotaxis	0.00	0.11		Pentose phosphate pathway	0.05	−0.04
Meso	Amino sugar and nucleotide sugar m.	0.05	−0.06		ABC transporters/PTS sys.	0.03	−0.04
	ABC transporters/Bacterial secretion sys.	0.04	−0.07		Alanine, aspartate, glutamate/Tryptophan m.	0.03	−0.05
	Galactose m./Starch and sucrose m.	0.03	−0.10		Nitrogen m.	0.06	−0.05
	Lysine degr./Tryptophan m.	0.04	−0.16		Glycerophospholipid m.	0.05	−0.06
	Glycerophospholipid m.	0.03	−0.17		Sulfur m.	0.01	−0.07
	Polyketide sugar b./Streptomycin b.	0.01	−0.35		Glutathione m.	0.02	−0.09
	Ascorbate and aldarate m.	0.01	−0.50		B. of unsaturated fatty acids	0.03	−0.13
**SEED**							
Thermo	Coenzyme M	0.00	2.46	Post	Coenzyme M	0.02	0.50
	Phage family-specific subsys.s	0.01	0.78		B. of galactoglycans, lipopolysacharides	0.02	0.05
	Hyp. associated with RecF	0.03	0.77		ATP synthases	0.05	0.04
	Bacteriocins, antibacterial peptides	0.02	0.57		Dormancy and sporulation	0.05	0.01
	Spore DNA protection/Dormancy, sporulation	0.00	0.35	Pre	Phosphorus M.	0.04	−0.03
	Flagella protein/Motility and Chemotaxis	0.01	0.32		Nitrogen M.	0.01	−0.04
Meso	Fatty acids, Lipids, Isoprenoids	0.05	−0.03		M. of aromatic compounds/M. of central aromatic intermediates	0.02	−0.05
	Electron donating	0.04	−0.05		Protein folding	0.04	−0.06
	Protein m./Protein b./Arginine; urea cycle, polyamines/Ammonia as.	0.02	−0.09		Stress response/Oxidative stress/Osmotic stress/Cold shock	0.03	−0.08
	Carbohydrates/Di- and oligosaccharides/Polysaccharides	0.02	−0.10		Sulfur M./Organic sulfur as.	0.02	−0.08
	Stress response/Osmotic stress/Heat shock	0.03	−0.13		Virulence, Disease and Defense/Invasion and intracellular resistance	0.03	−0.09
	Resistance to antibiotics and toxic compounds/Detoxification	0.02	−0.15		Motility and chemotaxis/Flagella protein	0.03	−0.09
	Capsular, extracellular polysacchrides/B. of galactoglycans, lipopolysacharides	0.01	−0.21		Potassium m.	0.01	−0.11
	Protein folding/Protein processing and modification/Two related proteases	0.03	−0.27		Two related proteases	0.03	−0.12
	Phosphorus m./Potassium m.	0.00	−0.33		Protein secretion sys.	0.04	−0.15
	Phospholipids/Fatty acid cluster/Hyp. lipase related to phosphatidate m.	0.03	−0.41		Lipids, isoprenoids/Phospholipids/Fatty acid cluster/Hyp. Lipase	0.02	−0.19
	Membrane-bound hydrogenase/Probably Ybbk-related hyp. membrane proteins	0.03	−0.45		Branched-chain amino acids/Glutamine, glutamate, aspartate/Ammonia as./Lysine b.	0.03	−0.24
	Phages, Prophages, Transposable elements/Phages, Prophages/CRISPRs	0.03	−0.49		Electron accepting/ Cytochrome biogenesis/Riboflavin	0.04	−0.55
	Adhesion	0.01	−0.67		Central carbohydrate m./Coenzyme B	0.04	−0.78

*KEGG and SEED functional categories were analyzed in order to evaluate their statistically significant effect on operational parameters (PrF indicate p-value), i.e., temperature and H_2_ addition. The log ratios of values obtained for PGs abundances under thermophilic or mesophilic conditions (left) and “post/pre” H_2_ addition (right) are considered as dependent variables. Parameter “b” is the regression coefficient of the target category (e.g., riboflavin metabolism), expressed as number of genes, on the dependent variable. The p-value refers to the effect of a target category on the dependent variable, with p = 0.05 as threshold for statistical significance. m, metabolism; b, biosynthesis; degr, degradation; sys, system; as, assimilation; hyp, hypothetical*.

The external H_2_ provision increased the H_2_ concentration in the liquid phase of the reactor, which in turn affected discordantly the abundance of PGs, potentially inhibiting SAO, promoting homoacetogens, or leaving unchanged the abundance of other species (i.e., in PGs that can possibly act both as SAOB and homoacetogens) (Table 2 and Figure 3). The bacteria that were negatively affected by H_2_ addition were *Syntrophomonadaceae* sp. DTU232, which had the most acute decrease in abundance (25-fold), *Clostridia* sp. DTU204 and *Tepidanaerobacter* sp. DTU063, which decreased 8 and 5-fold, respectively (Table 2 and Figure 3). On the contrary, *Clostridia* sp. DTU077, DTU122, and DTU183 were strongly favored by the presence of H_2_ (Table 2 and Figure 3). Among them, *Clostridia* sp. DTU183 was the most abundant and its change in abundance was significantly correlated with acetate concentration in the reactor before and after H_2_ addition (*p* = 0.026; Tables 1, 2). From a thermodynamic point of view, the increased H_2_ partial pressure can inhibit SAO since the syntrophic sustainability relies on the H_2_/formate concentration, which is maintained low by the methanogenic partners (Stams and Plugge, 2009). Once H_2_ content is intentionally increased, its utilization as electron donor for the reduction of CO_2_ to acetate would be more favorable. Thus, considering their similar gene content and opposite changes in abundance, DTU232, DTU204, DTU063, and DTU021 could most probably be classified as SAOB, and on the contrary DTU077, DTU122 and DTU183 are more preferable to act as homoacetogens. Other PGs (i.e., DTU027, DTU095, and DTU093) were more stable in abundance changing <1.5 fold after H_2_ addition, suggesting that they are able to follow the WL pathway in both directions or can shift to a different metabolism (Table 2 and Figure 3).

To drive these endergonic reactions, PGs can perform electron confurcation coupled with electron bifurcation, employing energy from oxidation of a low-potential donor and reduction of a high-potential acceptor (Sieber et al., 2012). Interestingly, most of PGs having WL pathway, did not have genes related to menaquinone biosynthesis (i.e., only three of them have one gene) indicating that these microbes use different processes to transfer H_2_ to methanogenic archaea. For example, the metabolic reconstruction analysis demonstrated that four PGs have energy conserving hydrogenase (ferredoxin) and five PGs have numerous genes involved in biogenesis of c-type cytochrome (Table 2 and SEED annotation on www.biogasmicrobiome.com under the “SYMBIO project”). The presence of these genes is a good indicator for syntrophic capacity in terms of bi-confurcation mechanisms but metatranscriptomics coupled with stable isotope probing (SIP) investigation are needed to further elucidate the process. One example is the recent finding of *Syntrophomonas* syntrophic LCFA-degraders in mesophilic anaerobic digesters, involving interspecies electron transfer (hydrogen and/or formate) by formate dehydrogenases and hydrogenases (Ziels et al., 2018).

### Influence of microbial variation on propionate and butyrate

Exploration of the PGs' metabolism provided fundamental insights into species involved in propionate and butyrate degradation. In addition to the previously discussed potential SAOB, 21 PGs were predicted to be involved mainly in butyrate and/or propionate degradation (i.e., KEGG Propanoate and Butanoate metabolism; Table 2 and Supplementary Data 4). Especially, six PGs were particularly enriched in propionate-related genes (mainly mesophilic), four PGs were rich in butyrate-related genes (mainly thermophilic) and the remaining PGs were predicted to be both propionate and butyrate degraders (propionate/butyrate). It is known that species belonging to *Syntrophobacterales* (e.g., *Syntrophobacter fumaroxidans*) use sulfate as the electron acceptor for propionate oxidation; in addition, they can grow by fermentation of pyruvate and fumarate (McInerney et al., 2008; Plugge et al., 2011). *Gammaproteobacteria* sp. DTU260 and *Firmicutes* sp. DTU245 were the propionate-specific degraders found in the mesophilic community and they showed opposite changes in abundance after H_2_ addition (Supplementary Data 2). While a decreased population is expected considering syntrophic attitude in response to enhanced H_2_ concentration, the case of *Firmicutes* sp. DTU245 is particularly interesting, being strongly favored by the excess of H_2_. In particular, it is worth noting that two common features among PGs favored by H_2_ excess are the presence of genes forming an alternative path for propionate degradation (i.e., 2-oxoisovalerate dehydrogenase and dihydrolipoamide dehydrogenase) and a high number of genes assigned to TCA cycle (Figure 3 and Supplementary Data 4). While the presence of DTU245 can explain the relatively low level of propionate in the mesophilic reactor, the absence of a corresponding functional redundant microbe can justify the accumulation of propionic acid (i.e., more than 5 fold increment) in the thermophilic reactor after H_2_ addition (Table 1).

Regarding butyrate metabolism, it is known that some *Syntrophomonadaceae* (e.g., *Syntrophomonas wolfei*) can perform β-oxidation. Moreover, the re-oxidation of reducing equivalents generated during this process lead to the production of H_2_ or formate (Wallrabenstein and Schink, 1994; McInerney et al., 2008). Generally, the butyrate-specific degraders tend to increase in abundance after H_2_ addition; particularly *Syntrophothermus* sp. DTU052 is one of the most interesting cases, possessing also nine isoforms of formate dehydrogenase (*fdh*) gene (Table 2). Accordingly, the concentration of butyrate in both temperature conditions decreased after H_2_ addition (Table 1).

Considering the propionate/butyrate degraders, *Alcaligenaceae* sp. DTU041 and *Corynebacterium* sp. DTU130 were inhibited by H_2_ addition, while *Proteobacteria* sp. DTU251 and *Syntrophomonadaceae* sp. DTU190 were favored (Table 2). *Corynebacterium* sp. DTU130 was found to be closely related to a known species, having a >97% ANI with *Corynebacterium humireducens* str. DSM 45392 (Supplementary Data 2), isolated from the anode of a wastewater-fed microbial fuel cell (Wu et al., 2011). However, the higher abundance of DTU130 at thermophilic condition compared to mesophilic together with the optimal growth condition of DSM45392 at 37°C are suggesting that they may have different preferences in growth temperature. *Proteobacteria* sp. DTU251 and DTU248 were assigned to *Alcaligenaceae* family (80% ANI with *Oligella ureolytica*) together with DTU041 and DTU260, and they were found to encode key proteins involved in sulfate reduction (e.g., sulfate adenyltransferase; Table 2 and SEED annotation on www.biogasmicrobiome.com under the “SYMBIO project”). This suggests their ability to use sulfate as the electron acceptor for propionate oxidation or for sulfate-dependent interspecies H_2_ transfer. Moreover, almost all 12 propionate/butyrate degraders have genes involved in menaquinone biosynthesis or binding (Table 2 and Supplementary Data 7). It is known that the conversion of malate to oxaloacetate (for propionate) and the oxidation of hydroxybutyryl-CoA (for butyrate) in obligate syntrophic microbes require input of metabolic energy (Figure 3). This process is fundamental for the formation of syntrophic interactions with the archaea and is coupled to menaquinone-mediated interspecies electron transfer between membrane complexes during metabolites oxidation (Sieber et al., 2012; Kougias et al., 2016).

Finally, it should be underlined that H_2_ addition had no univocal effect on propionate and butyrate degraders (Table 2 and Supplementary Data 2), which were regulated by specific syntrophic interactions with archaea. It can be hypothesized that only the obligate syntrophic microbes (following linear metabolism) miss an alternative strategy to compensate the negative impact of increased H_2_ partial pressure. Meanwhile, facultative syntrophic acidogens, which degrade sugars, or aminoacids and are following branched metabolism, are more flexible and can adjust their metabolism using alternative pathways.

## Conclusions

Genome-centric metagenomics applied to biogas upgrading reactors revealed for the first time insights into the metabolism of CO_2_ hydrogenation both at mesophilic and thermophilic conditions. It was demonstrated that the higher operating temperature decreased the microbial richness, while after addition of H_2_ the microbial compositions changed into different consortia more specialized on biomethanation process. Furthermore, the increased H_2_ partial pressure affected differently the abundance of the mesophilic and thermophilic microbial communities deranging the equilibrium of specific catabolic pathways. In particular, it was indicated that the metabolic flexibility of previously uncharacterized syntrophic bacteria could support potential activity as SAOB and also as homoacetogens. Finally, it was demonstrated that even though genomes closely related and assigned to crucial taxa for the specific bioprocess (e.g., *Methanoculleus* spp.) co-exist in the same ecosystem, they possess different metabolic features. Interestingly, despite the presence of a composite hydrogenotrophic archaeal population in the reactors, only one member was found to be dominant at each temperature condition, and thus, the main responsible for conversion of CO_2_ and H_2_ to methane.

## Availability of data and material

Sequence data used in this study were deposited at the National Center for Biotechnology Information (NCBI) as part of the BioProject PRJNA283612. Raw sequence data can be found at Sequence Read Archive (SRA) under accession SRP058235 and whole metagenome assembly is at DDBJ/EMBL/GenBank under the accession number LSQX00000000. All data and bioinformatics procedures can be found at www.biogasmicrobiome.com.

## Author contributions

LT designed experiment, analyzed the metagenomic data, reconstructed the metabolic pathways and wrote the manuscript. SC designed experiment, analyzed metagenomic data and wrote the manuscript. PK designed experiment, interpreted the results and revised the manuscript. CS performed the statistical analysis and revised the manuscript. IB monitored the reactors operation, analyzed the biochemical data and revised the manuscript. IA designed experiment and revised the manuscript.

### Conflict of interest statement

The authors declare that the research was conducted in the absence of any commercial or financial relationships that could be construed as a potential conflict of interest.

## Abbreviations

AD: Anaerobic Digestion
ANI: Average Nucleotide Identity
Cov: Coverage
CSTR: Continuous Stirred Tank Reactor
KO: KEGG Orthology
Mcov: Mean Coverage
PG: Population Genome
SIP: Stable Isotope Probing
SAO: Syntrophic Acetate Oxidation
SAOB: Syntrophic Acetate Oxidizing Bacteria
SD: Standard Deviation
TCA: Tricarboxylic Acid
WL: Wood-Ljungdahl.
