# Supplementary material for: Hydrogen-Fueled Microbial Pathways in Biogas Upgrading Systems Revealed by Genome-Centric Metagenomics
Source: Front Microbiol. 2018 May 28;9:1079. doi: 10.3389/fmicb.2018.01079 (PMC5985405; doi:10.3389/fmicb.2018.01079)
Supplement: Supplementary file 1 [file Data_Sheet_1.docx]

Supplementary Material

Hydrogen-fueled microbial pathways in biogas upgrading systems revealed by genome-centric metagenomics

**Laura Treu^1^ , Stefano Campanaro^2^, Panagiotis G. Kougias^1^*, Cristina Sartori ^3^, Ilaria Bassani^1^ and Irini Angelidaki^2^**

^1^ Department of Environmental Engineering, Technical University of Denmark, Kgs. Lyngby DK-2800, Denmark

^2^ Department of Biology, University of Padova, Via U. Bassi 58/b, 35131 Padova, Italy

^3^ Department of Agronomy Food Natural Resources Animals and Environment (DAFNAE), University of Padova, Viale dell'Università 16, Legnaro, PD 35020, Italy

* **Correspondence:** Panagiotis G. Kougias: [panak@env.dtu.dk](mailto:panak@env.dtu.dk)

Hereafter supplementary comments are reported concerning PGs taxonomic assignment, variation in abundance and details on putative functional roles of the microbial species.

**Microbial taxa with temperature-specific abundance profiles**

The general microbial community was investigated with different statistical methods (clustering analysis, single standard deviations from the whole population mean value, fold changes), applied to the population genomes (PG) abundance values at mesophilic and thermophilic conditions. Results from all the analyses indicated that the community structure differed significantly in terms of abundance profile mainly due to the operational temperatures. Therefore the PGs were assigned to mesophilic or thermophilic community according to their abundance profiles.

Additional clustering analysis was performed considering differences in PGs abundance due to the H_2_ injection (P=0.03). The obtained result was similar for the two communities: 41% of the PGs decreased more than 2-fold in thermophilic conditions and 38% in mesophilic (P=0.30). On the contrary, H_2_ addition resulted in the increased abundance of some specific PGs, and this was more evident on the mesophilic community (13% in thermophilic and 29% in mesophilic conditions; P<0.001) (Supplementary data 2). According to these results, the number of PGs assigned to specific taxa was calculated separately for mesophilic and thermophilic communities, before and after external H_2_ injection (Figure S1).


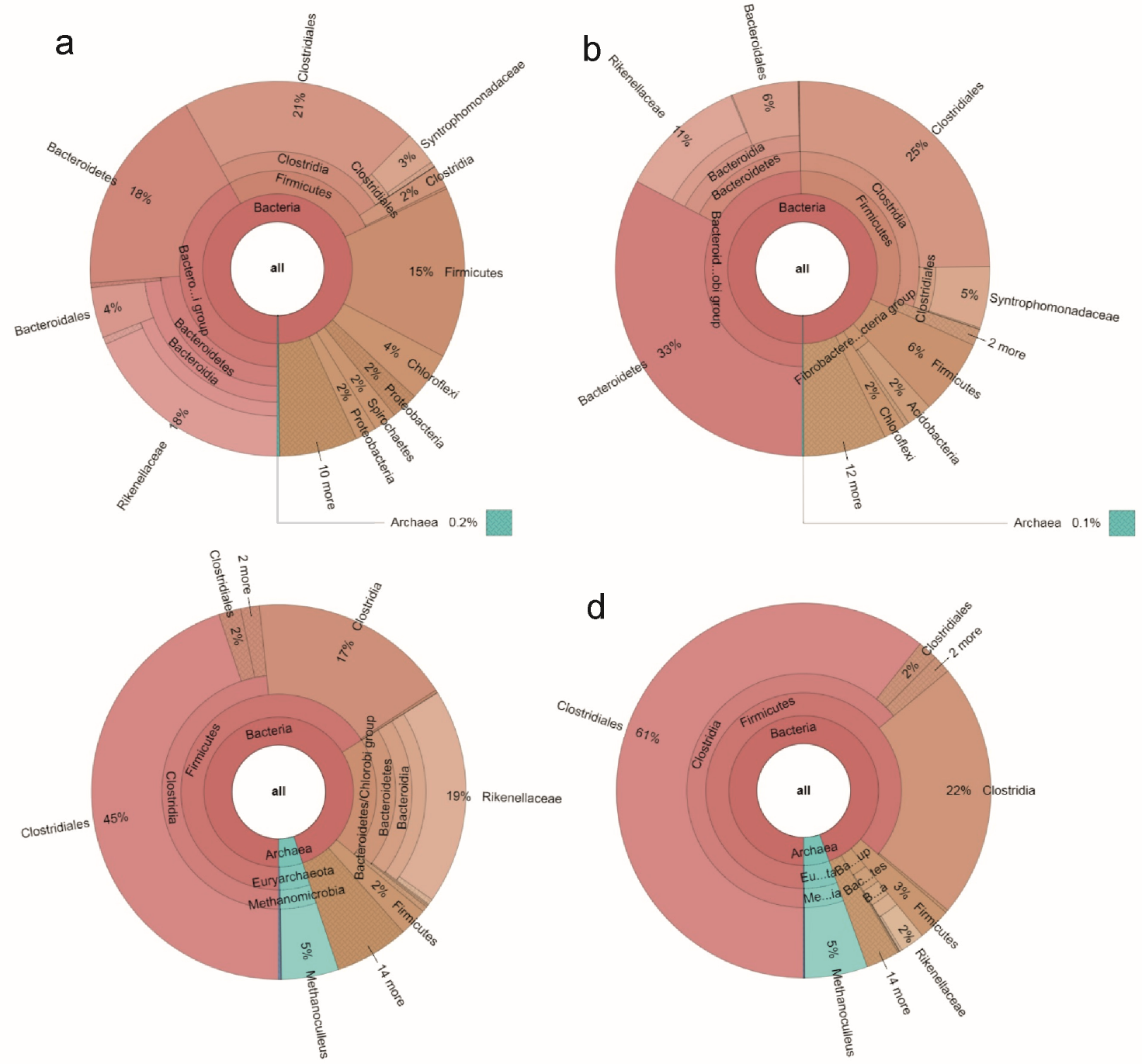


**Supplementary Figure 1.** Krona plots of microbial community. Effects of process conditions, i.e. temperature and H_2_ addition, on taxonomies of the identified PGs. Plots represent numerical count of PGs in (A) mesophilic reactor before H_2_ addition, (B) mesophilic reactor after H_2_ addition, (C) thermophilic reactor before H_2_ addition, (D) thermophilic reactor after H_2_ addition.

Among the most represented phyla, 23 PGs were assigned to *Proteobacteria*. As a general trend, upon H_2_ addition, they decreased on average 3-fold. Conversely, three PGs showed the opposite behavior, increasing up to 95-fold (*Xanthomonadaceae* sp. DTU255, *Proteobacteria* sp. DTU174 and *Proteobacteria* sp. DTU168). The increase of these last two *Proteobacteria* spp. was >3SD of PGs overall mean, that includes 99% of values. Members of family *Pseudomonadaceae*, phyla *Actinobacteria* and *Tenericutes* showed a concordant decrease after H_2_ addition and are known to be involved in recalcitrant compounds decomposition producing enzymes for lignocellulose degradation (Boucias et al., 2013; Ntougias et al., 2013). The most represented phylum in both mesophilic and thermophilic communities is *Firmicutes* with 141 PGs, and this is determined by the numerous fundamental activities performed in the AD microbiome, where they perform polysaccharides and proteins hydrolysis, acetate synthesis and syntrophic VFA degradation (Krakat et al., 2011). *Erysipelotrichaceae* family is present in both temperature conditions in low abundance and is negatively influenced by H_2_, with six of eight PGs assigned to *Erysipelothrix rhusiopathiae*. The highest number of microbes in the AD community was assigned to *Clostridiales*, 126 PGs, with 22% of them evenly distributed among the two temperature conditions and not being influenced by H_2_ presence (with only few exceptions).

Considering *Firmicutes* phylum, *Clostridiales* sp. DTU175 was the only member of *Thermoanaerobacteraceae* and the third most abundant in the mesophilic community (up to 98 cov), favored by H_2_ addition. *Tenericutes* phylum was found to be represented by five PGs, generally present at low abundance and decreasing upon H_2_ addition. The huge decrease in abundance of DTU186 was the most significant of the community, but the metabolic reconstruction of this PG was not possible due to the high number of unknown proteins in its genome. Other two *Firmicutes* PGs belonging to *Thermoanaerobacteraceae* were mainly found in the thermophilic community, and in particular *Clostridia* sp. DTU011 was the third most abundant microbe of the community. A functional reconstruction of this PG highlighted the presence of several different groups of transporters, specific for amino acids, sugars, lipids and metals (63 genes), suggesting its ability to uptake a huge variety of substrates (Supplementary data 4). Among *Halanaerobiaceae*, two PGs were found to be thermophilic and, particularly, *Halothermothrix* sp. DTU029 was positively correlated to H_2_ addition increasing its abundance from 5.6 up to a coverage value of 13. DTU029 possesses 22 genes related to cellular response to stress and it was potentially classified as protein utilizer, with 54 genes assigned to amino acid metabolism and transport (Supplementary data 4). The only mesophilic PG belonging to *Erysipelotrichaceae* is *Firmicutes* sp. DTU224, highly abundant before H_2_ (22 cov) but strongly inhibited after H_2_ addition (>5-fold). *Erysipelotrichaceae* might be involved in acetic and propionic acid degradation (Stolze et al., 2015). The 27% of all *Clostridiales* were mainly mesophilic (34 PGs), with six PGs being highly abundant (>8 cov) in at least one condition. In particular, *Syntrophomonadaceae* sp. DTU220 and *Clostridiales* sp. DTU225 were strongly increased in abundance (8 and 5-fold, respectively) and *Firmicutes* sp. DTU245 (discussed in detail in the next sections) and *Clostridiales* sp. DTU230 were weakly increased (2 and 1-fold), indicating a positive correlation of their abundance with H_2_ concentration in the reactor. Conversely, *Firmicutes* sp. DTU246 showed an opposite behavior, strongly decreasing after H_2_ addition by 5-fold. Moreover two less abundant PGs showed opposite and extreme reactions to H_2_ presence, *Syntrophomonadaceae* sp. DTU218 increasing in abundance 37-fold and *Firmicutes* sp. DTU206 decreasing 49-fold (resp. >2 SD and <2 SD of the PGs overall mean for pre/post H_2_).

Considerations regarding other phyla included *Proteobacteria* and *Verrucomicrobia*. Most of the PGs assigned to *Proteobacteria* were mesophilic and five out of nine were negatively influenced by H_2_ addition. This is probably due to their relevant role in H_2_ production, for this reason a detailed discussion of some crucial species such as *Alcaligenaceae* sp. DTU041 and *Gammaproteobacteria* sp. DTU260 is reported in main manuscript. Moreover, all the *Verrucomicrobia* PGs were present at low abundance with DTU166 presenting the higher increase upon H_2_ addition (<3-fold). Despite DTU166 is taxonomically related to the methanotrophic *Methylacidimicrobium tartarophylax* (van Teeseling et al., 2014) having 90% similarity by 16S rRNA gene BLAST, it seems unable to utilize methane as energy source; in fact it lacks proteins used for methane oxidation (e.g. pMMO, methane monooxygenase) suggesting a completely different functional role, yet to be elucidated.

Even if all PGs were identified at both temperature conditions, statistical analysis indicated that 38 PGs had less than 2-fold abundance difference between the two temperature conditions, all of them having less than five coverage values (Supplementary data 2). The four PGs belonging to *Actinobacteria* are generally rare (mean cov <2) and decreasing after H_2_ addition (4-fold on average). *Lentisphaerae* phylum was represented by *Lentisphaerae* sp. DTU164 only, and it was positively affected by H_2_ addition (up to 5-fold increase). Members of this phylum were previously isolated from anaerobic granular sludge (e.g. *Oligosphaera ethanolica* 8KG-4) (Qiu et al., 2013) but up to now very few genomes have been analyzed. Functional annotation of this genome confirms its ability to import and ferment monosaccharides, like fructose and galactose (*via* PTS-system and homoacetogenesis) and indicates a possible involved in polysaccharides degradation. Despite it was found able to produce H_2_, we did not identified membrane-bound cytochromes, menaquinones and ferredoxins indicating absence of H_2_ transfer to syntrophic H_2_-scavenging species.

Among rare phyla (mean cov <0.9), *Synergistetes* was represented by seven PGs, whose abundance did not markedly differ between the two temperature conditions (with the only one exception previously described). An interesting finding was the high similarity of *Synergistaceae* sp. DTU120 with *Aminobacterium colombiense* str. DSM 12261 (99.5% ANI). The low abundance of this PG and the general decrease of this phylum after H_2_ addition (3-fold average) can be explained by *A. colombiense* ability in H_2_ production. In fact, the higher H_2_ partial pressure might have played a suppressive effect on these microorganisms (Chertkov et al., 2010). The functional properties of *A. colombiense* are confirmed in DTU120 by the numerous genes involved in AA transport (14 genes in phosphate and amino acid transporters; polar amino acid transporter) and utilization (32 genes in “amino acid utilization biosynthesis metabolism - glycine, serine and threonine metabolism”). Results obtained from KEGG indicate also its acetogenic behavior (11 genes), while SEED revealed a high number of membrane-bound hydrogenases (nine genes) (Supplementary data 4 and 6). Similarly, phylum *Verrucomicrobia* was found in very low abundance and represented by three PGs, two of which were common to the two temperature conditions.

**Dominant mesophilic archaea**

As reported in the main manuscript, the dominant archaea of mesophilic community was a hydrogenotrophic *Methanoculleus* species, different from the thermophilic *Methanoculleus* DTU006. The reconstructed PG (defined as “*Methanoculleus* spp. DTU000”) was not fulfilling the quality criteria of the current study, since it had high contamination level, and thus, it was excluded from the official set of reconstructed PGs. The abundance of *Methanoculleus* spp. DTU000 was calculated as average coverage of the scaffold assigned to this PG (Figure S2).


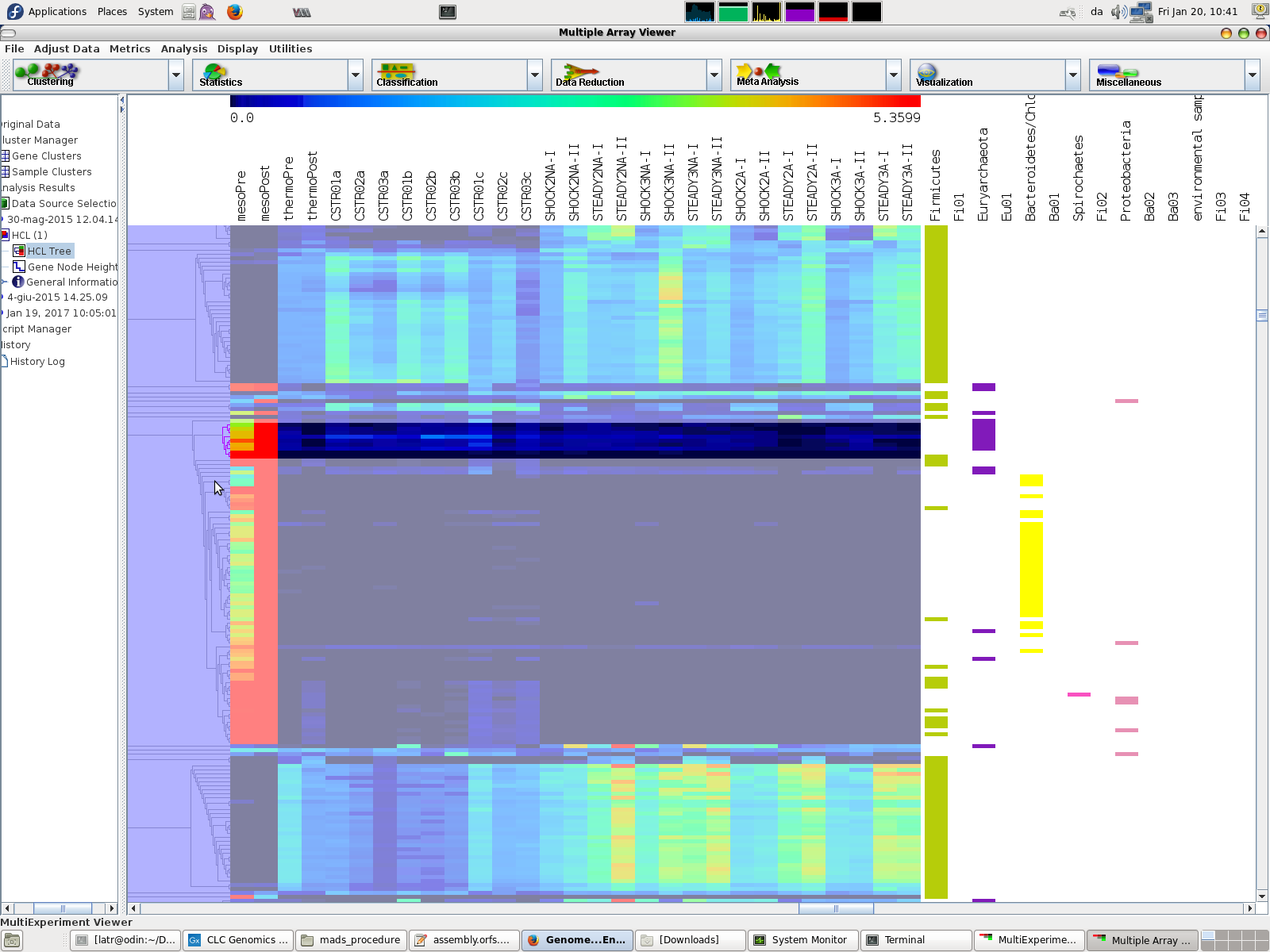


**Supplementary Figure 2.** Binning of archaeal genome DTU000. The presence of DTU000 in high abundance in the mesophilic (first two columns) biogas microbial community has been evidenced during the binning procedure.

**Effect of KEGG and SEED functional categories on operational parameters**

A specific analysis was performed in order to highlight functional categories with variation in gene number that significantly affect (α=0.05) the PGs abundance in mesophilic vs. thermophilic conditions and before vs. after H_2_ addition. The regression coefficient (*b*) determined comparing the number of genes in a target functional category with the PGs abundance in “thermophilic/mesophilic condition” and “post/pre H_2_” addition was reported (Supplementary data 7). An example of functional category significantly higher in thermophilic environment is the Coenzyme M (SEED; *b*= 2.46; P<0.001; Supplementary data 7), implied in methyl-transfer reactions of methanogenic archaea (Hallam et al., 2003). As previously reported, archaeal PGs found in reactors are more abundant in thermophilic conditions, and examples of PGs are *Methanoculleus thermophilus* DTU007 and *Methanothermobacter* sp. DTU051.

An interesting category is related to spore DNA protection/dormancy and sporulation (SEED; *b*= 0.35; P<0.001), since the higher temperature may cause an increased production of spores and an augmented dormancy of microorganisms to cope with the hard environmental conditions (Gould, 2006). The SEED category dormancy and sporulation was largely found in other studies, and mainly associated with *Clostridia* and *Bacilli* taxa (Mury and Popham, 2014; Singh et al., 2014). An increased number of genes involved in riboflavin metabolism was found under thermophilic conditions (KEGG; b=0.26; P=0.04; Supplementary data 4). Riboflavin (vitamin B2) plays an important role in the basic metabolism of many microorganisms, being a precursor of coenzymes flavin adenine dinucleotide (FAD) and flavin mononucleotide (FMN), and being involved in a number of extracellular processes such as extracellular electron transfer (Vitreschak et al., 2002).

Considering the functional categories affecting the variation of PGs abundance after H_2_ addition (Supplementary data 7), a significant increase in the number of genes after addition was found for the SEED categories of Coenzyme M (*b*= 0.50; P=0.02) and dormancy and sporulation (*b*=0.01 and P=0.05), also significant under thermophilic environment, as reported before. After the H_2_ addition some functional categories related to metabolism had a slight but significant increase in the number of genes, i.e. the polyketide sugar unit biosynthesis (KEGG; *b*= 0.12; P=0.04), the unit of biosynthesis of galactoglycans and related lipopolysaccharides (SEED; *b*= 0.05; P=0.02), and the ATP synthases (SEED; *b*= 0.04; P=0.05). Polyketides are a wide range of secondary metabolites produced by various microorganisms to receive some survival advantage (Helfrich et al., 2014). It is possible to hypothesize that polyketides offer some advantages in an environment under selective pressure. Glycans (including galactoglycans) are involved in a number of important physiological processes, comprising cell recognition, adhesion, signal transduction, and quality control (Tanaka, 2016). The SEED category of biosynthesis of galactoglycans and related lipopolysaccharides during increased H_2_ environments has not been investigated yet, but this category has been already found in microbial communities of some extreme environments, such as in snow samples from Artic seasonal snowpack, where it plays a role in cold resistance (Maccario et al., 2014), and in *Actinobacteria* of the anoxic sediments of the Baltic Sea deepest point (Thureborn et al., 2016).

A number of functional categories has been also found to have a statistically significant increased number of genes before the H_2_ addition (Supplementary data 7). These categories cover a number of metabolic pathways; an example is the biosynthesis of unsaturated fatty acids (KEGG; *b*=-0.13; P=0.03); the addition of H_2_ enhance the conversion into saturated fatty acids, requiring free hydrogen and NADPH. Other categories related to microbial metabolism showing a significant greater number of genes after H_2_ addition are Fatty Acids, Lipids, and Isoprenoids/Phospholipids/Fatty acid metabolic cluster/Hypothetical lipase related to Phosphatidate metabolism/Triacylglycerols (*b*=-0.19; P=0.02) and Electron accepting reactions/Cytochrome biogenesis/Riboflavin, FMN, FAD/Putative Isoquinoline 1-oxidoreductase subunit (*b*= -0.55; P=0.04).

**Other Supplementary Materials for this manuscript includes the following:**

**Supplementary data 2.** Taxonomy and coverage values obtained for all the PGs of the community. Details on different methods used for taxonomy assignment are reported for each PG as follows: (A) Univocal name of PG used in the manuscript; (B) ID of PG as reported in the manuscript; (C) Genome size [bp] estimated considering the total length of scaffolds assigned to PG; (D) number of genes identified using Prodigal run in metagenomic mode in the global assembly and assigned to each PG considering the binning results; (E) Total number of essential genes identified in the PG; (F) Univocal essential genes identified in the PG; (G) Completeness estimated considering the total number of essential genes of the PG compared to the total number of essential genes of the corresponding phylum (in green are highlighted values higher than 90%); (H) Genome duplication estimated considering the univocal number of essential genes and the total number of essential genes (including multiple copies, in green are highlighted values lower than 5%); (I-O) Domain, phylum, class, order, family, genus and species determined by Phylophlan; (P) ANI - match with NCBI genome; (Q) ANI - value [%]; (R) Common essential genes [%]; (S) 16S rRNA gene BLAST best match; (T) 16S rRNA gene BLAST Identity [%]; (U) 16S rRNA gene BLAST coverage [%]; (V) 16S rRNA gene localization [scaffold]; (W) 16S rRNA gene size [bp]; (X) Coverage of PG estimated at mesophilic condition before H_2_ addition; (Y) Coverage of PG estimated at mesophilic condition after H_2_ addition; (Z) Coverage of PG estimated at thermophilic condition before H_2_ addition; (AA) Coverage of PG estimated at thermophilic condition after H_2_ addition.

**Supplementary data 3.** Maximum likelihood tree of *hmrA* gene with support values in Newick format. The tree reports the archaeal PGs of the AD upgrading community together with archaeal genomes deposited at the NCBI and having the *hmrA* gene. Suggested viewer: FigTree http://tree.bio.ed.ac.uk/software/figtree/

**Supplementary data 4.** Number of genes belonging to KEGG pathways identified in the PGs and statistical over-representation for each KEGG pathway. Details on the KEGG annotation for each PG are described as follows: (A) Univocal name of PG used in the manuscript; (B) ID of PG as reported in the manuscript; (C) number of genes identified using Prodigal run in metagenomic mode in the global assembly and assigned to each PG considering the binning results; (D-FL) number of genes identified in each KEGG pathway; (FO-LW) p-value for over-representation of the genes in each KEGG pathway obtained from 1000 random resampling performed on all the genes assigned using the binning strategy (nd=not determined).

**Supplementary data 5.** Number of genes belonging to COG categories identified in the PGs and statistical over-representation for each COG category. Details on the COG annotation for each PG are described as follows: (A) Univocal name of PG used in the manuscript; (B) ID of PG as reported in the manuscript; (C-X) number of genes assigned to each COG category; (Z-AU) p-value for over-representation of the genes in each COG category obtained from 1000 random resampling performed on all the genes assigned using the binning strategy (nd=not determined).

**Supplementary data 6.** Number of genes belonging to SEED categories identified in the PG. Details on the SEED annotation for each PG are described as follows: (A) Univocal name of PG used in the manuscript; (B) ID of PG as reported in the manuscript; (C-GX) number of genes identified on each SEED category. In the first line (C1-CX1) are reported the names of the SEED categories and in the second line (C2-CX2) is reported the annotation level considered (FL=first level; SL=second level).

**Supplementary data 7.** Statistics related to coverage values of the PGs (sheet “standard deviations”) and regression analysis between PGs abundance in the different operational conditions and functional categories of KEGG (sheet “reg KEGG”) and SEED (sheet “reg SEED”). Details on the statistical values obtained for each PG in sheet “standard deviations” are reported as follows: (A) Univocal name of PG used in the manuscript; (B) ID of PG as reported in the manuscript; (C) Completeness of PG; (D-I) transformation of coverage data into pairwise comparisons using log for each condition, as described in the Methods, (D) Thermo post/pre H_2_, (E) Meso post/pre H_2_, (F) Mean post/pre H_2_, (G) Pre_H_2_ thermo/meso, (H) Post_H_2_ thermo/meso, (I) Mean thermo/meso; (J-O) One standard deviation of single PGs from the whole population mean abundance value, - and + ; (P-U) Two standard deviations of single PGs from the whole population mean abundance value; (V-AA) Three standard deviations of single PGs from the whole population mean abundance value. Results of multiple regression analysis performed on functional categories with a significant impact on the relative abundance of PGs in sheet “reg KEGG” and “reg SEED” are reported as follows: (A) comparison of the operational conditions under investigation; (B) functional category with significant effect on operational parameters; (C) significance (p-value) of the effect of a target category on the dependent variable; (D) regression coefficient of the target category on the dependent variable.

References

Boucias, D. G., Cai, Y., Sun, Y., Lietze, V. U., Sen, R., Raychoudhury, R., et al. (2013). The hindgut lumen prokaryotic microbiota of the termite *Reticulitermes flavipes* and its responses to dietary lignocellulose composition. *Mol. Ecol.* 22, 1836–1853. doi:10.1111/mec.12230.

Chertkov, O., Sikorski, J., Brambilla, E., Lapidus, A., Copeland, A., Glavina Del Rio, T., et al. (2010). Complete genome sequence of *Aminobacterium colombiense* type strain (ALA-1). *Stand. Genomic Sci.* 2, 280–9. doi:10.4056/sigs.902116.

Gould, G. W. (2006). History of science – spores. *J. Appl. Microbiol.* 101, 507–513. doi:10.1111/j.1365-2672.2006.02888.x.

Hallam, S. J., Girguis, P. R., Preston, C. M., Richardson, P. M., and DeLong, E. F. (2003). Identification of methyl coenzyme M reductase A (*mcrA*) genes associated with methane-oxidizing archaea. *Appl. Environ. Microbiol.* 69, 5483–5491. doi:10.1128/AEM.69.9.5483-5491.2003.

Helfrich, E. J. N., Reiter, S., and Piel, J. (2014). Recent advances in genome-based polyketide discovery. *Curr. Opin. Biotechnol.* 29, 107–115. doi:10.1016/j.copbio.2014.03.004.

Krakat, N., Schmidt, S., and Scherer, P. (2011). Potential impact of process parameters upon the bacterial diversity in the mesophilic anaerobic digestion of beet silage. *Bioresour. Technol.* 102, 5692–5701. doi:10.1016/j.biortech.2011.02.108.

Maccario, L., Vogel, T. M., and Larose, C. (2014). Potential drivers of microbial community structure and function in Arctic spring snow. *Front. Microbiol.* 5. doi:10.3389/fmicb.2014.00413.

Mury, S. P., and Popham, D. L. (2014). Bacterial Endospores. *eLS*, 168–172. doi:10.1002/9780470015902.a0000300.pub2.

Ntougias, S., Bourtzis, K., and Tsiamis, G. (2013). The microbiology of olive mill wastes. *Biomed Res. Int.* 2013. doi:10.1155/2013/784591.

Qiu, Y. L., Muramatsu, M., Hanada, S., Kamagata, Y., Guo, R. B., and Sekiguchi, Y. (2013). *Oligosphaera ethanolica* gen. nov., sp. nov., an anaerobic, carbohydrate-fermenting bacterium isolated from methanogenic sludge, and description of *Oligosphaeria classis* nov. in the phylum *Lentisphaerae*. *Int. J. Syst. Evol. Microbiol.* 63, 533–539. doi:10.1099/ijs.0.039545-0.

Singh, K. M., Reddy, B., Patel, A. K., Panchasara, H., Parmar, N., Patel, A. B., et al. (2014). Metagenomic analysis of buffalo rumen microbiome: Effect of roughage diet on Dormancy and Sporulation genes. *Meta Gene* 2, 252–268. doi:10.1016/j.mgene.2014.01.005.

Stolze, Y., Zakrzewski, M., Maus, I., Eikmeyer, F., Jaenicke, S., Rottmann, N., et al. (2015). Comparative metagenomics of biogas-producing microbial communities from production-scale biogas plants operating under wet or dry fermentation conditions. *Biotechnol. Biofuels* 8, 14. doi:10.1186/s13068-014-0193-8.

Tanaka, K. (2016). Chemically synthesized glycoconjugates on proteins: effects of multivalency and glycoform in vivo. *Org. Biomol. Chem.* 14, 7610–7621. doi:10.1039/C6OB00788K.

Thureborn, P., Franzetti, A., Lundin, D., and Sjöling, S. (2016). Reconstructing ecosystem functions of the active microbial community of the Baltic Sea oxygen depleted sediments. *PeerJ* 4, e1593. doi:10.7717/peerj.1593.

Vitreschak, A. G., Rodionov, D. A., Mironov, A. A., and Gelfand, M. S. (2002). Regulation of riboflavin biosynthesis and transport genes in bacteria by transcriptional and translational attenuation. *Nucleic Acids Res.* 30, 3141–51.
